# Herd Immunity to Fight Against COVID-19: A Narrative Review

**DOI:** 10.7759/cureus.33575

**Published:** 2023-01-09

**Authors:** Yasha N Suryawanshi, Dalia A Biswas

**Affiliations:** 1 Physiology, Jawaharlal Nehru Medical College, Datta Meghe Institute of Medical Sciences, Wardha, IND

**Keywords:** covid-19, reinfection, mutation, immunocompromised, vaccine, herd immunity, coronavirus

## Abstract

The advent of severe acute respiratory syndrome coronavirus 2 (SARS-CoV-2) and its consequent illness, coronavirus disease 2019 (COVID-19), has revealed the severe impact of new, contagious pathogens on the population throughout the globe. Here, we describe the fundamental notions of herd immunity and discuss their consequences from the perspective of COVID-19, along with the obstacles to acquiring herd immunity. SARS-CoV-2 causes COVID-19, a contagious respiratory infection. It is a major global health issue, with more than 179 million positive cases and 3.8 million deaths globally. It has impacted more than 159 countries; hence, the World Health Organization designated it a pandemic. Different vaccines have been developed against coronavirus to slow the spread of this deadly virus. Immunizing people against coronavirus is the key to getting through this infectious virus. The central concept of this review article is the effect of vaccinating a large population to achieve herd immunity and the reasons for the delay in developing herd immunity. Herd immunity can prove highly beneficial for dealing with reinfection. Moreover, it can reduce the severity of the reinfection in many people who are twice infected with COVID-19. Herd immunity can prevent people in the high-risk group such as immunocompromised individuals; those on immunosuppressants; organ transplant recipients; particular age groups such as neonates, infants, toddlers, and elderly; those with impaired immunity; those with anaphylaxis reactions; and people with chronic diseases. However, due to repeated mutations of the virus, it is evolving into new strains with more severity. Its consequences on the immune system and response to a vaccine are still a big challenge to overcome. How new variants of COVID-19 impacted herd immunity needs to be investigated. The duration required for the development of herd immunity and how long it would last is still under research, along with the number of doses needed, booster doses, and the proportion of the population to be vaccinated.

## Introduction and background

The recent global pandemic, coronavirus disease 2019 (COVID-19), caused by the highly virulent severe acute respiratory syndrome coronavirus 2 (SARS-CoV-2), is extremely contagious, rapidly spreading, and is the cardinal cause of increased morbidity and mortality rates worldwide [1]. Remarkable attempts are being made to develop efficient and safe vaccines to prevent pathogen spread, reduce infection severity, and develop an immune response against COVID-19. Vaccinating a vast population to establish herd immunity, reduce the severity of infection, and safeguard high-risk groups and immunocompromised individuals is essential [2]. This review article determines the effects of herd immunity on COVID-19 and the hurdles in acquiring herd immunity, the duration to attain herd immunity, the vaccine dose required, and the booster dose. If most of the people in society are vaccinated, herd immunity can develop, which will prevent the severe effect on individuals who are suffering from allergies to vaccines, neonates, individuals on immunosuppressants and organ transplantation, and individuals with certain diseases, such as cancer, cardiac conditions, mental illness, acquired immunodeficiency syndrome. However, mutations in COVID-19 affect herd immunity and the response of vaccines to new variants. Several vaccines are being studied or have been licensed for use in emergencies globally. SARS-CoV-2 immunity to minimize the spread of COVID-19 or the severity of illness is necessary [3]. Herd immunity works by obtaining a population-level barrier immunity which is capable of hypothetically cutting the transmission chain of a specific infectious disease, achieved by natural infection or vaccination. However, the latter would undeniably depend on the durability of individual natural or vaccine-induced immunity. Nucleic acids, viral vectors (replicating and non-replicating), virus-like particles, peptide-based, recombinant proteins, live-attenuated, and attenuated virus modalities have all been used to produce vaccine candidates. Although several extensive clinical trials evaluating the clinical effectiveness and safety of COVID-19 vaccinations have reported encouraging results, many of these vaccines are still in preclinical testing [4].

## Review

Methodology

We performed a systematic search through PubMed and PubMed Central in August 2022 using keywords such as “covid-19” and “herd immunity” (((covid-19 [Title/Abstract]) OR (coronavirus [Title/Abstract])) OR (mutation*[Title/Abstract])) OR (“covid-19” [MeSH Terms]) AND ((“herd immunity” [Title/Abstract]) OR (immunocompromised [Title/Abstract])) OR (“herd immunity” [MeSH Terms]). We additionally searched for key references from the bibliographies of the relevant studies. The search was updated in October 2022.

**Figure 1:**
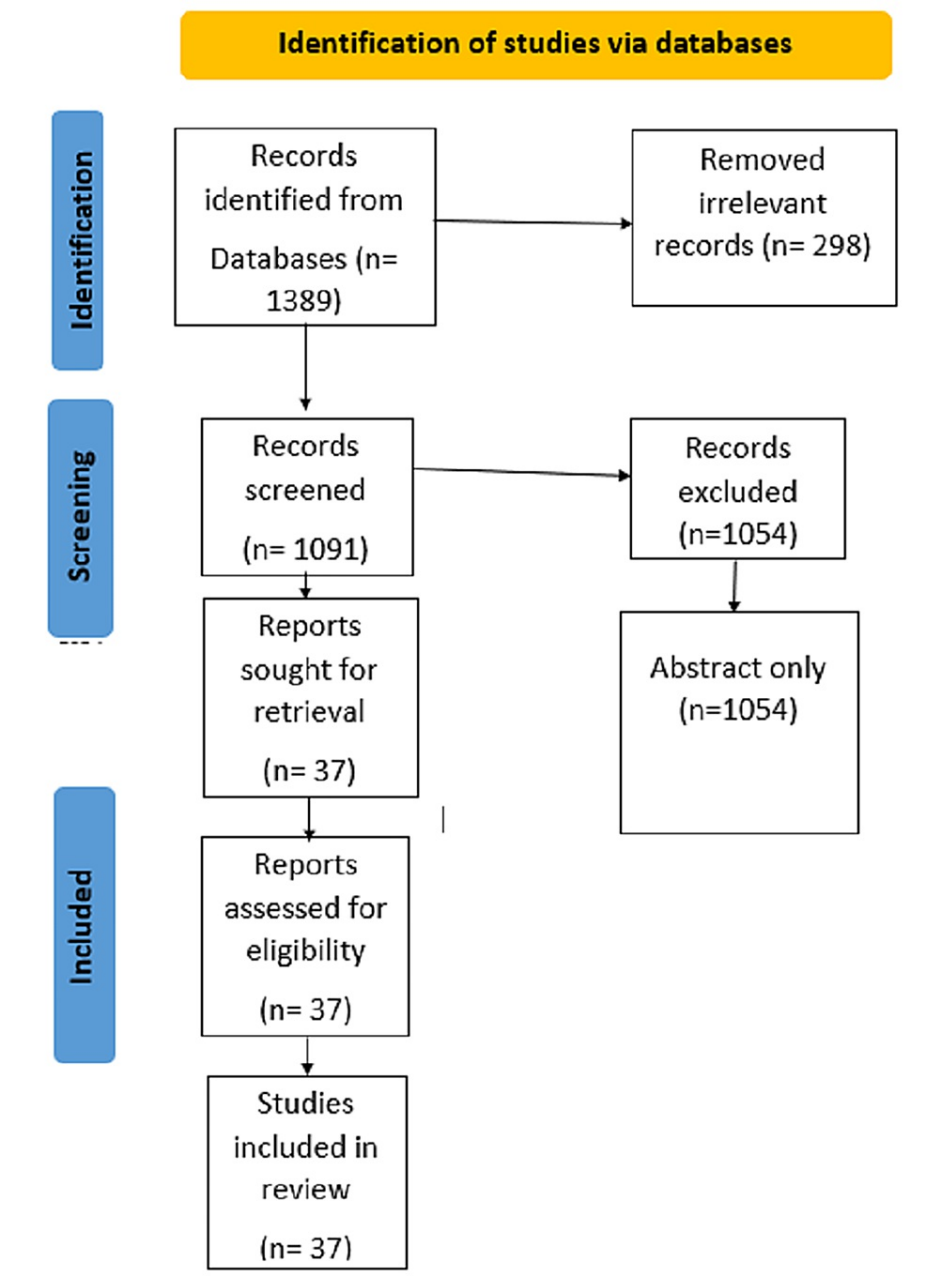
Preferred Reporting Items for Systematic Reviews and Meta-Analyses flowchart.

One reviewer independently monitored the retrieved studies against the inclusion criteria based on the title and abstract and then on full texts. Another reviewer also reviewed approximately 20% of these studies to validate the inclusion of studies. Data were extracted and tabulated. Details about the percentage of the population vaccinated with single, double, and booster doses and some variants of the COVID-19 study were tabulated. Figure 1 shows the number of records identified from each database or registered searched, as well as the records that were excluded.

Herd immunity, also called community immunity, is when a significant fraction of the population is immune to a specific pathogen and develops resistance against a particular virus or bacteria, following which the infection rate falls, and the disease dwindles [5]. It can be acquired in two ways, either by infection with the pathogen or vaccination. Using safe and effective vaccines inducing herd immunity reduces disease prevalence and decreases mortality and morbidity. Herd immunity not only protects an individual but the community as a whole is protected [4]. If herd immunity is achieved, it can safeguard vulnerable groups, including people at higher risk [6]. Herd immunity is determined by R_0_, which refers to the average number of secondary infections caused by an infected person. Consider a hypothetical pathogen with an R_0_ of 4, on average, one infected person will infect four others during the exposure, assuming no immunity exists in the population. Mathematically, the herd immunity threshold is defined by 1 - 1/R_0_, and R_0_ is directly proportional to the contagious tendency of COVID-19 [7]. For a massive spread of disease, a considerable proportion of the community must be susceptible to it. This population is considered the threshold proportion [8]. If the number of people immune to the disease is higher than the specific immunity threshold, the disease outspread slows and falls, which is known as the herd immunity threshold. Herd immunity requires 75-85% of the population to be vaccinated, and the more the intensity of infection, the more the population needs to be vaccinated [9]. The main barrier to acquiring herd immunity is that SARS-CoV-2 is undergoing frequent mutations in its spike protein, causing changes in its genome sequence and resulting in various modifications in the virus [10]. Antiviral vaccine generation focuses on structural proteins such as glycoprotein, spike protein, and amino acid sequencing. It might help in discovering compounds with antiviral capabilities; however, the high ability for the sudden mutation associated with its genetic properties, such as increased recombination capacity and presence of a splicing site and more than 40 RNA modification sites, might pose difficulty because it can provoke changes in the specificity of specific tissues and antiviral sensitivity [11]. Accordingly, the methodology for developing vaccines becomes more demanding and challenging to achieve enough efficiency to oppose the new variant. Furthermore, several severe issues exist regarding the generation of herd immunity in COVID-19 [12]. Table 1 shows the percentage of the population vaccinated with single, double, and booster doses. Several vaccine doses administered also affect the duration of gaining herd immunity [13].

**Table 1:** Data of vaccine doses administered.

Name of country	Total vaccine doses administered per 100 population	Persons fully vaccinated with the last dose of primary series per 100 population	Persons boosted per 100 population
India	142.35	65.85	3.93
United States	176.98	66.32	31.26
Japan	224.51	80.9	61.44
Italy	229.6	79.61	68.01
Africa	61.98	34.26	5.79

The effect of the number of vaccine doses and booster doses on herd immunity, according to some researchers, takes at least six months to develop immunity after administering a vaccine, and the duration up to which immunity will last remains unknown [14].

Major hurdles in acquiring herd immunity

Vaccine-hesitant people are not getting vaccinated because they fear their hazardous effects, and some have religious beliefs regarding COVID-19. There is much fake news about COVID-19 vaccines on various social media platforms. Some people blindly follow it and spread it in their community [15]. People already suffering from certain diseases such as cancer, cardiovascular diseases, and diabetes are hesitant to take the vaccine. They are afraid to take vaccines because of specific health issues. Moreover, uneven vaccine access due to inadequacy or supply in remote areas is a big challenge for various countries. Vaccination for children has been delayed because most vaccine trials have been conducted on people over the age of 18 [16]. Pregnant women were also concerned about taking vaccines, especially those with complicated pregnancies. Persons suffering from mental issues were also concerned about getting vaccinated [17]. All these conditions significantly contribute to the delay in crossing threshold immunity. Indeed, the World Health Organization (WHO) and United Nations International Children’s Emergency Fund are involved after new data show that the overall immunization program kept dwindling in 2021, with 25 million infants lacking life-saving vaccines, according to the WHO data published on July 15, 2022. Because COVID-19 is affecting other global vaccination campaigns for children due to the massive spread of such a contagious virus, parents are restricted from taking their children out for various other vaccine campaigns. There is a need to conduct a thorough study in the field of immunity toward COVID-19 to tackle this problem.

COVID-19: vaccine allergy and effects on immunocompromised people

Herd immunity against COVID-19 can rescue people who suffer from allergies to COVID-19 vaccines. In these people, taking the vaccine can cause anaphylaxis, polyethylene glycol allergy, and polysorbate allergy [18]. COVID-19 vaccine impacts immunocompetent patients, cancer patients, transplant recipients, human immunodeficiency virus patients, and those receiving immunomodulatory therapy for autoimmune disorders, such as rheumatoid arthritis and myasthenia gravis, multiple sclerosis, and others due to impaired immune response [19]. Cancer patients and solid-organ transplant recipients may be more liable to severe COVID-19 disease, which means if such people are infected with COVID-19, they can suffer from severe complications. However, if a large population is immunized, the chances of person-to-person COVID-19 transmission decrease [8]. Most people infected with COVID-19 inculcate an immune response within the first few weeks of infection and the strength of protection, and how long it would last remains under research. Moreover, in case of reinfection, the severity of the disease would be mild to moderate after sufficient doses. It can also reduce post-COVID-19 complications such as loss of smell and taste, weakness, mucormycosis, blood clots, and muscle pain. Moreover, research is ongoing regarding the effect of immunization on post-COVID-19 or long-term COVID-19 infections [20]. SARS-CoV-2 elicits different immune responses in different people depending on the severity of the symptoms. It is unclear whether people who recovered from COVID-19 have adequate immunity to prevent reinfection.

Immunology

The determination of the SARS-CoV-2 immunological memory state aids in the identification of reinfection risk and vaccination efficacy [21]. As a result, upon recovery from COVID-19, an evaluation of the protective illness and long-term immunity from past infection can be crucial [22]. Current studies have characterized the kinematics of SARS-CoV-2-specific cellular and humoral responses in recuperating SARS-CoV-2 patients for more than six months [23]. According to available information, natural killer (NK) cell subpopulations, particularly the memory-like NK cell subset, play a crucial part in defining COVID-19 seriousness. The data on long-term NK cell immunity imparted by SARS-CoV-2 infection are limited [24]. Results from vaccination trials and observation and resulting studies suggest that naturally occurring immunity, induced immunity, and combined immunity to severe acute respiratory coronavirus emerge as particularly effective [25]. Most people are asymptomatic or have mild symptoms, which begs the question of how there is a difference in the generation of immune response in patients with critical illness [26]. In the early infective stages, dysregulated effector immune cells, such as neutrophils, macrophages, monocytes, megakaryocytes, basophils, eosinophils, and erythroid progenitor cells, and Th17 cells might influence a patient’s progression to severe disease. However, adequately functioning CD4+, CD8+, NK cells, and DCs minimize the serious consequences of the disease. A thorough study of generating an immunological response in recuperating patients migrating from the effector phase to the immunogenic memory phase can provide critical clues to identifying critical variables to evaluate.

The first-generation COVID-19 vaccinations were potent in preventing acute illness and hospitalization. Still, recurrent episodes of infections are associated with SARS-CoV-2 variants that exhibit an increased ability to evade antibodies, resulting in decreased vaccine effectiveness [27]. Evasive COVID-19 variants represent a crucial knowledge gap in our search for broadly protective vaccinations. Effective respiratory or systemic CD4 and CD8 T memory cells protected against coronavirus variants can be elicited in the presence or absence of virus-neutralizing antibodies using adjuvanted spike protein-based vaccines. Immunity due to mucosal memory CD8 T cell was mainly redundant in the presence of antibodies that efficiently neutralized the challenge virus, and unhelp mucosal memory CD8 T cells provide little protection against the homologous SARS-CoV-2 in the absence of CD4 T cells and neutralizing antibodies. Thus, inducing systemic and mucosal memory T cells directed against conserved epitopes can be an effective method for protecting against immune-evading SARS-CoV-2 variants [28]. Despite advances in our understanding of SARS-CoV-2 immunological memory, understanding how these responses translate into protection against reinfection at both the individual and population levels remains a big problem. Extraordinarily protective and lasting immunity allowing the formation of increased levels of herd immunity will be an absolute total result following infection or immunization. Neutralizing antibody responses in recovering patients are declining. Recorded incidences of SARS-CoV-2 reinfection are also increasing. Anticipating the dynamic aspect of memorizing answers to COVID-19 and immune control procedures is critical for rational vaccine design and deployment and understanding possible future pandemic trajectories. As of now, four COVID-19 variants of concern, B.1.1.7, B.1.351, B.1.617.2, and P.1, as well as two mutants of interest, C.37 and B.1.621, are identified to potentially escape immunity, and greater than two or more mutations and evolution provide them with troubling epidemiological, immunological, or pathological features, as presented in Table 2 [29].

**Table 2:** Some variants of COVID-19.

WHO label	Pango lineage	GISAID clade
Omicron	B.1.1.529	GR/484A
Alpha	B.1.1.7	GRY
Beta	B.1.351	GH/501y.v3
Delta	P.1	
Gamma	B.1.617.2	GR/501Y.V3

Rapid population immunity by vaccinating a large population is required to prevent mutations and establish different variations, which can entirely evade immune scrutiny. COVID-19 vaccinations are harmless and can significantly decrease the number of deaths, critical case scenarios, cases showing significant symptoms, and the spread of infection due to COVID-19 globally [30]. In respect of a worldwide pandemic and the proceeding arrival of COVID-19 mutants, rapidly immunizing and enhancing immunization proportion remains a highly essential and pressing issue and the only way to halt the epidemic. Double doses of the COVID-19 vaccination significantly reduced hospitalization, complicated cases, and deaths due to COVID-19 [31].

The worldwide spread of numerous COVID-19 mutations calls into question whether current vaccines will give long-lasting protection. A vaccine generating responsive antibodies against receptor-binding protein comparable to naturally occurring infection implies that vaccines can, to some extent, lessen the degree of disease produced by natural conditions. Furthermore, mutations have little effect on T-cell responses to spikes, which means that variants can still induce serious illnesses. Again, as coronavirus transmutation continues to develop, the body’s immune response is continuously building its ability to deal with the mutant changes in the COVID-19 virus. After 6.2 months of successful immunization, memory B cells are constantly developing and are engaged in preventing reinfection. This information strongly impacts the fact that vaccinated people can respond swiftly and efficiently when exposed to the virus. It is revealed that various present messenger ribonucleic acid, adenovirus, and deactivated vaccine doses can elicit a noteworthy immunological response in contrast to SARS-CoV-2 receptor-binding domains in vaccine recipients. Individuals develop antibody responses against receptor-binding domains after 30 days of receiving both immunization doses. COVID-19 vaccines are safe like other vaccines, such as ribonucleic acid, adenovirus family, and deactivated vaccinations, and are similarly satisfactory compared to various vaccines, including the influenza vaccine [32].

Because COVID-19 immunization rates have gone up worldwide, people wonder how prolonged this epidemic will exist. It is a question fraught with ambiguity. However, the assumption that sufficient people will ultimately develop antibodies to COVID-19 to preclude a major herd immunity threshold is becoming doubtful. Such a criterion is generally attained through elevated vaccination rates. Several researchers believed that once people began to get vaccinated in large numbers, population immunity would help society revert to normal. Several analysts predict the criterion at 60-70% of the community acquiring immunity from immunization or recent infection with the pathogen. However, while the epidemic approaches its second wave, perceptions are shifting. Youyang Gu, an autonomous data scientist, updated the title of his renowned COVID-19 forecasting model from “Path to Herd Immunity” to “Path to Normality” in February. He stated that attaining a population immunity threshold seems implausible owing to characteristics such as vaccine reluctance, the advent of novel variations, and the late advent of child vaccines [33].

To achieve the aim of reducing and eliminating COVID-19, herd immunity must fulfill the requirements. The accomplishment of herd immunity is hardly possible unless any of the causes for the herd immunity concept is established [34]. Three significant challenges in herd immunity including novel variations, irregular vaccine distribution globally, and immunized people’s behavioral changes must be resolved. A new vaccination must be capable of dealing with novel variations with spike mutations and antigenic escape, along with unequal distribution of vaccines across the globe. Vaccinated people’s alterations in behavior add to the prevalence of contagious diseases. Currently, as COVID-19 cases are not rising, many people have started ignoring precautions and guidelines for the prevention of COVID-19. This kind of behavior also plays a role in the delay of population immunity [35].

Different assumptions made by researchers regarding herd immunity

To acquire population immunity to resist SARS-CoV-2, Anthony Fauci anticipated that it is essential to vaccinate 70-85% of the US population [36]. Population immunity is a hypothesis built on statistical models instead of coherent reasoning [37]. Datasets require a variety of conditions to be fulfilled. For example, within these estimates, the vaccine’s efficacy must remain constant.

## Conclusions

Building herd immunity will reduce the probability of infection with COVID-19. In addition, it will significantly reduce the severity of reinfection and hospitalization. However, the duration to achieve herd immunity highly depends upon vaccination, completing the required dose, including booster doses, and controlling mutations. Worldwide continuous analysis of SARS-CoV-2 variants and their virulent impact will allow scientists to determine whether to update vaccines and other treatments. Newly emerging variants may diminish herd immunity, threaten unvaccinated people, and promote vaccination escape, predisposing people to severe illness or death. Most research has shown that immunizations are effective. Vaccine development for COVID-19 has advanced at an unprecedented rate in the last year, which was not previously conceivable. Achievement of herd immunity is a challenging idea to grasp. In global immunization, many countries are arranging vaccination campaigns, and vaccines have been administered exponentially.
